# Involvement of Subinsular Territory Stroke as Predictor of Outcome after Successful Endovascular Recanalization of Left Middle Cerebral Artery Occlusion

**DOI:** 10.3390/brainsci14090885

**Published:** 2024-08-30

**Authors:** Yasuyuki Yoshida, Tatsushi Mutoh, Yasuko Tatewaki, Yasuyuki Taki, Junta Moroi, Tatsuya Ishikawa

**Affiliations:** 1 Department of Surgical Neurology, Research Institute for Brain and Blood Vessels, Akita Cerebrospinal and Cardiovascular Center, Akita 010-0874, Japan; 2 Department of Aging Research and Geriatric Medicine, Institute of Development, Aging and Cancer, Tohoku University, Aoba-ku, Sendai 980-8575, Japan

**Keywords:** acute ischemic stroke, clinical outcome, endovascular therapy, futile recanalization, middle cerebral artery, subinsular territory

## Abstract

Subinsular stroke (subIS) can occur between the penetrating middle cerebral artery (MCA) branches, which clinical and radiological findings sometimes encounter in patients after the recanalization of left proximal MCA occlusion. However, no supportive data are available to clarify this relationship. This study investigated whether the involvement of subIS can impact outcomes after successful reperfusion therapy. Data from 152 consecutive patients who underwent endovascular thrombectomy between 2019 and 2023 were collected. A 3-month functional independence defined as a modified Rankin Scale 0–2 (primary outcome) and influencing factors were analyzed retrospectively. Recanalization was achieved in 35 patients, of whom 11 (31%) developed subIS. Patients with subIS were older in age (81 vs. 75; *p* < 0.05), had lower apparent diffusion coefficient (ADC) values on admission (0.52 vs. 0.62; *p* < 0.001), and higher modified Rankin Scale (mRS) scores (4 vs. 2; *p* < 0.001) than those without subIS. In a multivariate analysis, subIS was independently associated with a worse functional outcome (odds ratio: 10.5, *p* = 0.02). The cut-off value of the ADCs was 0.52 with a sensitivity and specificity of 70% and 64%, respectively. Subinsular ischemic lesions contribute to poor functional independence in patients after the successful recanalization of left MCA occlusion. The attenuation of the ADC value in these territories could be a valuable predictor of the outcome.

## 1. Introduction

Stroke ranks as the second leading cause of death and permanent disability worldwide. Throughout Asian countries including Japan, about 70% of strokes are caused by acute ischemic stroke (AIS) [1]. Reperfusion therapy using intravenous tPA thrombolysis and endovascular thrombectomy (EVT) has been the standard treatment of AIS due to large-vessel occlusion [2]. Despite the therapeutic benefit of EVT, which is associated with high rates of the recanalization (71–84%) of occluded vessels, more than half of patients still do not achieve favorable outcomes [3].

Futile recanalization without functional independence, defined as the proportion of patients who do not benefit from EVT recanalization, has received increasing attention from physicians and rehabilitation therapists. Currently, several studies focusing on the mechanisms and predictors of futile recanalization have been conducted to explore effective diagnostic and interventional approaches for better prognosis [4,5]. In a recent multicenter study, old age, high National Institutes of Health Stroke Scale (NIHSS) scores, and failure of complete recanalization were shown to be independently associated with futile recanalization [6].

In terms of functionality, the left (dominant) hemisphere is more important than the right. Accumulating evidence suggests that patients with proximal anterior circulation occlusion in the left side have severe stroke on admission and a half of them result in poor outcomes after EVT recanalization, despite increased rates of functional independence at 3 months [7]. In fact, we have previously reported a case of successful recanalization after EVT recanalization for left middle cerebral artery (MCA) occlusion by demonstrating the impairment of subcortical territories related to neural network and cellular viability in the arcuate fasciculus using diffusion MR fiber tractography and benzodiazepine receptor imaging [8]. However, only limited data are available for assessing the specific area(s) responsible for the white matter and/or penetrating small-vessel injury after futile recanalization. Although a specific region has not been detected, atrophic changes affecting the temporal and parieto-occipital regions, as well as a higher degree of cortical and subcortical atrophy, have shown to be associated with futile recanalization in patients with proximal anterior circulatory occlusions [9]. Several clinical and imaging studies support that collateral circulation status may be linked to poor functional outcomes after successful recanalization [10,11]. On the other hand, there are conflicting data suggesting the occlusion location that involves subcortical white matter arising from the precentral gyrus (particularly on the left side), and the posterior limp of the internal capsule of white matter corticospinal tracts can be preserved by post-EVT recanalization, irrespective of the collateral status [12]. Taken together, we postulate that brain regions between the deep and superficial MCA territories and related junctional areas (i.e., internal border zone or watershed) [13], with limited collateral reserve supplied by small perforating arteries [14], are particularly susceptible to ischemic infarction, and thus may constitute a predictor of the patient outcome after successful reperfusion therapy.

Cerebral infarcts extending to the subinsular region, so-called subinsular stroke (subIS), can occur in the deep border zone between the perforating branches of the lenticulostriate and insular penetrating arteries of the MCA. Typical clinical symptoms include transcortical motor aphasia and dysphagia, as well as motor and sensory disturbances [15], and about half of the patients suffer from severe residual deficits [16], all of which closely resemble complications following the recanalization of the proximal left MCA (M1) occlusion. SubIS deserves attention for its characteristic anatomic and prognostic implications, because it is different from ischemic strokes limited to the insular territory where the affected patients usually attain good functional recovery [17,18].

To fulfill the gaps between technical success and clinical outcomes, we set our focus on identifying the acute subinsular ischemic lesion in patients after the successful EVT recanalization of left M1 occlusion. Thus, this study aimed to investigate the impact of subIS involvement on clinical outcomes for functional independence and to pursue related early-imaging biomarker(s) as predictors of successful reperfusion therapy for left MCA occlusion, based on MR imaging data acquired in the super-acute phase.

## 2. Materials and Methods

### 2.1. Study Design and Patient Definitions

Data of all the consecutive patients who underwent EVT between January 2019 and December 2023 at the Akita Cerebrospinal and Cardiovascular Center were retrospectively retrieved. The eligibility criteria for this study were as follows: patients (1) who underwent acute initial magnetic resonance imaging (MRI) immediately after admission and were diagnosed with AIS; (2) in whom left proximal MCA occlusion by MR angiography was detected and confirmed by subsequent digital subtraction angiography; (3) who had a pre-morbid modified Rankin Scale (mRS) score 0–1; (4) who had a thrombus located on the M1 (horizontal) segment of the MCA and received EVT within 24 h from stroke onset to groin puncture; and (5) who showed successful recanalization defined as a modified treatment with a cerebral infarction (mTICI) score ≥ 2b after EVT and functional independence defined as a 3-month mRS ≤ 2 [19]. The exclusion criteria were (1) occlusion of posterior circulation; (2) tandem occlusions; and (3) failed EVT recanalization.

The study protocol was approved by the Ethics Committee of the Research Institute for Brain and Blood Vessels of the Akita Cerebrospinal and Cardiovascular Center (protocol code: 24-5). The need for written informed consent was waived because of the retrospective and observational nature of the study.

### 2.2. Data Collection

The following individual patient data with baseline characteristics and clinical information were obtained: age, sex, medical history (e.g., hypertension, diabetes, and atrial fibrillation), baseline blood pressure, baseline Alberta Stroke Program Early CT Score (ASPECTS), NIHSS score on admission, subgroup of unknown stroke onset (wake-up stroke and daytime-unwitnessed stroke), treatment with intravenous thrombolysis, etiology according to the Trial of Org 10,172 in Acute Stroke Treatment, EVT procedure, time from stroke onset to admission, stroke onset to recanalization, and puncture to recanalization. The patients were divided into two groups with and without an involvement of acute infarcts in the subinsular territory that occur in the external or extreme capsular region (see Supplementary File S1 for representative images) [20]. SubIS was defined visually by assessing high signal intensities on diffusion-weighted (DWI) and T2-weighted MR images (T2WI) on admission and at 24 h after EVT, and confirmed by a low-density area on computed tomography (CT) at 3 weeks, extending subjacent to the insular cortex that occupied at least one third of the anteroposterior portion (Figures S1 and S2) [15,16], which can be differentiated from the lesion restricted to the insular cortical/operculum and/or putaminal structures (Figure S3). The Siemens 3.0T scanning system (MAGNETON^®^ Skyra, Siemens Healthcare GmbH, Erlangen, Germany) was used for magnetic resonance imaging (MRI) data acquisition. For the DWI, single-time activated SE-EPI sequences were used with the following parameters: TR/TE = 5000 ms/57 ms, acquisition matrix 128 × 128, FOV 324 mm × 230 mm, with diffusion gradient in the x, y, and z dimensions, and acquired images at b = 0 and 1000. The T2WI was performed using TSE sequences with TR/TE = 4000 ms/93 ms; the acquisition matrix was 325 × 512. All scanning sections were 5 mm thick and 2 mm apart. 

**Figure 1:**
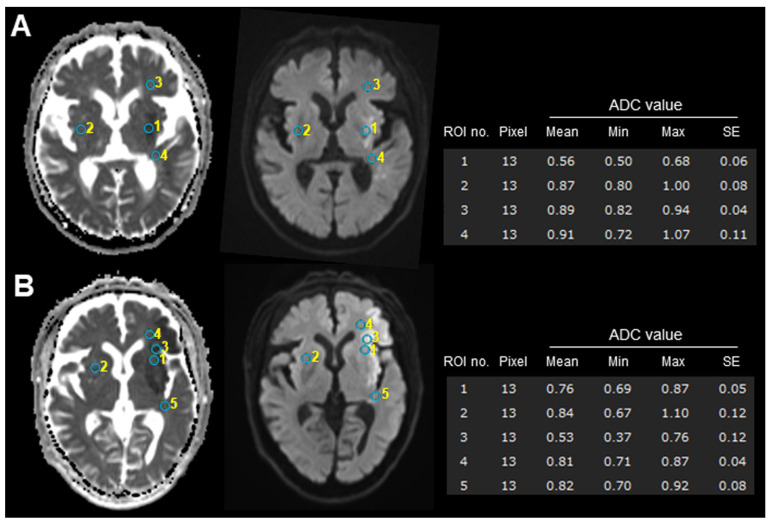
Examples of ROI measurement with (**A**) and without (**B**) subIS on admission MRI. Infarction areas reveal high signal intensities on diffusion-weighted MRI (**left**) and show low signal intensities on ADC map (**right**). ADC values of circular-shaped ROIs placed on subinsular territory (no. 1) in subIS (**A**) were lower than those of other case (**B**). Lower ADC values of infarcted areas (no. 1 in (**A**) and no. 3 in (**B**)) were observed, compared to adjacent subcortical white matter or contralateral side. See Supplementary Material for each patient detail. ADC, apparent diffusion coefficient; ROI, region of interest.

All MRI data were independently reviewed by two experienced radiologists who were blinded to the radiological and clinical outcomes. For quantitative analysis, apparent diffusion coefficient (ADC) maps were automatically calculated using a workstation (SYNAPSE VINCENT Ver.6.7, Fujifilum Co., Tokyo, Japan) (see Supplementary File S2 for representative images). Three regions of interest (ROIs) were selected from the central area of the ischemic lesion (Figures S4 and S5 for subIS, and Figure S6 for no-subIS), and the adjacent white matter in the frontal and temporal opercula and contralateral side (for reference) on the ADC figures according to the DWI and T2WI, with circular-shaped ROIs, were used to compute the average ADC value of the infarcted region (Figure 1).

### 2.3. Outcome Assessments

To evaluate neurological functional disability as a global outcome, the graded interval scales (ranging from 0 [no symptoms] to 6 [death]) of a 3-month mRS score were used. The primary outcome was a favorable outcome close to functional independence defined as an mRS 0–2 (poor outcome defined as ≥3). The secondary outcome measures were as follows: (1) early neurological improvement, which was defined as a decrease of 4 points or more in the NIHSS score at 24 h after receiving EVT compared with admission; and (2) symptomatic intracerebral hemorrhage.

### 2.4. Statistical Analysis

Data were presented as the means ± standard deviations or median with interquartile range if not normally distributed. Categorical variables were described as counts (%) and compared using Fisher’s exact test. Continuous variables with normal and non-normal distributions were compared using the *t*-test and Mann–Whitney U test, respectively. Multivariate logistic regression analysis was conducted to determine the independent predictors (including subIS and possible risk factors) of post-EVT recanalization after the adjustment of potential confounders (i.e., variables that revealed statistically significant differences between the subIS and non-subIS groups). The ability of substantial parameter(s) to predict the outcome was further determined using receiver operating characteristic curve analysis. A *p*-value of <0.05 was considered statistically significant. All analyses were performed using Bell Curve for Excel (Ver.4.07; SSRI, Tokyo, Japan), SPSS (Ver.28.0; IBM Corp., Armonk, NY, USA), and Prism version 10 (GraphPad Software, La Jolla, CA, USA). 

## 3. Results

### 3.1. Patient Characteristics

**Figure 2:**
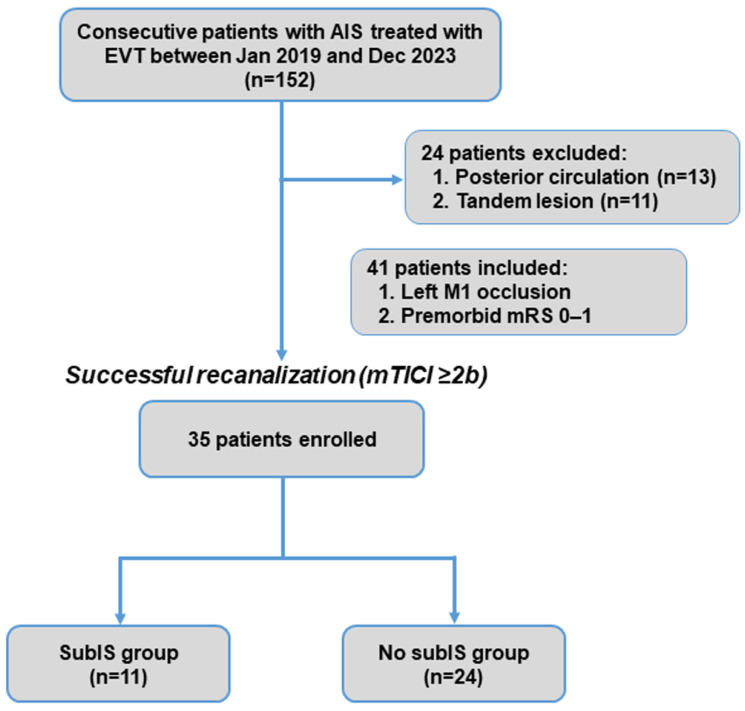
Patient inclusion flowchart. AIS, acute ischemic stroke; EVT, endovascular thrombectomy; mRS, modified Rankin Scale; subIS, subinsular stroke.

Participant enrollment is shown in Figure 2. In total, 152 consecutive patients with ischemic stroke who underwent EVT were enrolled in this retrospective cohort study (Table 1). A total of 24 patients were excluded due to posterior circulation (*n* = 13) or tandem (*n* = 11) occlusions. Of 128 patients with single-vessel occlusion of anterior circulation, 41 had left M1 occlusion, and 35 patients who had successful recanalization entered the study.

**Figure 3:**
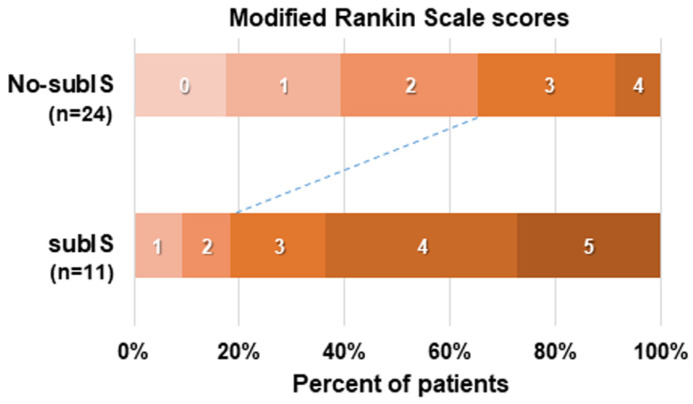
Functional outcome at 3 months in 35 patients with or without subIS, assessed using modified Rankin Scale scores.

The recanalization of the occluded vessel was achieved in 35 patients (85%) (Figure 2), of whom 11 (31%) involved a subIS, either restricted (n = 3) or coexisting ischemic lesion with adjacent insular cortex and/or basal ganglia (n = 8), and 24 had no-subIS on MRI images. In our study, the 3-month functional independence of patients with a subIS was lower than that of patients without a subIS (Figure 3). 

For typical prognostic symptoms, either motor (n = 3) or transcortical motor aphasia (n = 8) was observed in all patients with subIS. Recovery from right sensory and motor deficits, including hemiparesis and dysarthria, was variable, but gradually improved in eight cases to obtain walking abilities at 3 months. Persistent aphasia and executive control deficits (n = 7) appeared to interfere with the patients’ functional independence at the chronic stage (Figure 3). 

### 3.2. Clinical Outcomes and Risk Factors

Patients with subIS after successful recanalization were older (81 vs. 75 years, *p* < 0.05), had a lower ADC on admission (0.52 ± 0.08 vs. 0.62 ± 0.10 × 10^−3^ mm^2^/s, *p* < 0.001), and had a higher incidence of futile recanalization (81 vs. 33%, *p* = 0.01) associated with worse mRS scores (4 vs. 2, *p* < 0.001) than those without subIS (Table 1). No statistically significant differences between the groups with and without subIS in other clinical or laboratory data were observed.

**Figure 4:**
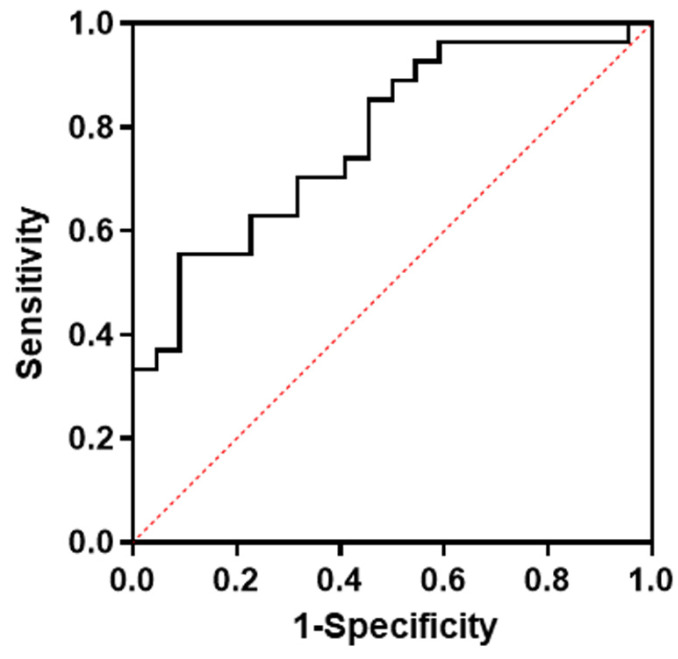
ROC curve for optimal cut-off levels of ADC values in subinsular territory on admission MRI. ADC, apparent diffusion coefficient; ROC, receiver operating curve.

Multivariate regression analysis including factors that may be related to futile recanalization after left M1 occlusion (i.e., age, initial NIHSS, and subIS) demonstrated that subIS was independently associated with unfavorable outcomes (odds ratio: 10.5 [1.53–72.3], *p* = 0.02) (Table 2). The optimal cut-off value of the ADC value in the subIS group was 0.52 × 10^−3^ mm^2^/s with an area under the curve of 0.78, and a sensitivity and specificity of 70% and 64%, respectively, were seen in predicting the 3-month functional outcome (Figure 4).

## 4. Discussion

In this study, we compared post-EVT recanalization patients with and without subIS involvement after the successful recanalization of left M1 occlusion and determined that the lesion could be an important predictor of 3-month functional independence. SubIS involvement was common, accounting for up to 30% of the patients. The early detection of decreased ADC values, in addition to physicians’ visual assessment of DWI signals, could be a valuable biomarker for poor prognosis.

Interestingly, such a small area may serve as a strategic hub for post-EVT neurological deterioration, even after the successful recanalization/reperfusion of large-vessel occlusion. Anatomically, subinsular territories (external capsular and/or the extreme capsular region) receive blood supply from the deep border zone between the branches encountering the lenticulostriate and insular penetrating arteries [16]. Although a definitive vascular etiology for subIS is not well defined, cardioembolism and the related hemodynamic mechanism have been thought to be a major potential cause [15,16]. Hypoperfusion in the cortical branches of the MCA due to the thrombi occluding the M1 segment may reduce collateral flow to the insular region, and thus result in focal infarction in a perisylvian area of decreased perfusion reserve and vulnerability to hypoperfusion injury after recanalization. Considering the lower oxygen consumption of the white matter (less than five times) than that of the gray matter [21], it is less reasonable to ascribe, even though the embolus is shorter, selective involvement to the subinsular white matter without corresponding changes in the striatum. The number of patients with restricted subIS without involvement of the insular cortex and striatum is very small (0.4%), and the majority (72%) are primarily affected by insular cortical infarcts or nearby large lenticulostriate infarcts, as demonstrated in our analysis.

What is the main reason for the poor functional independence in post-EVT recanalization patients with subIS? Clinically, the prognosis of restricted ischemic lesions in the subinsular territory is good [22,23]. However, to the best of our knowledge, only a few previous studies or case reports have described the clinical manifestations of subIS. Owing to the confluence of functions in a restricted region, subIS results in multimodal deficits combined in descending order of motor and somatosensory, speech or language, and cognitive disturbances. Although it did not appear in our patients, anterior opercular syndrome, characterized by a cortical type of pseudobulbar palsy and loss of voluntary movement of the orofacial and pharyngeal muscles with preserved automatic and reflex movements secondary to unilateral subcortical lesions, has also been described in previous reports [24].

Given that most of the prognostic symptoms were aphasia and impaired executive function, left insular and subinsular neural connectivity with other brain areas should be considered. Previous MRI-based structural studies have demonstrated the corticocortical connections of the insular cortex with the frontal, temporal, parietal, and occipital lobes [25]; however, only a few have focused on its relationship with subcortical territories. In the subinsular region, the arcuate fasciculus consists of various neural tracts related to language and cognitive functions. In a recent report, we have demonstrated the disrupted white matter tract integrity and neuronal viability of the arcuate fasciculus in a post-EVT patient after the successful recanalization of left M1 occlusion, using diffusion-tensor MRI fiber tracking and central benzodiazepine receptor SPECT imaging [8]. Furthermore, the anterior–superior aspect of the subinsular cortex involves the frontal aslant tract, a left predominant white matter front–frontal tract connecting the superior frontal gyrus originating at the pars opercularis that supports executive functions, such as attention and working memory over a range of language and speech processes [26]. Because older age is an additional prognostic factor for worsening language and cognitive functions, exploring the relationship between focal clinical symptoms and the ischemic injury of neural tracts in the subinsular cortex (see Figure S7 for representative images) may contribute to the establishment of more accurate predictors. However, at present, we believe that the ADC value and visual inspection of DWI signals on admission MRI would be the best choices for imaging biomarkers to assist clinicians in estimating the neural state, setting scientific rehabilitative strategies, and predicting the prognosis of post-EVT recanalization.

This study has several limitations to consider. First, this was a single-center, retrospective cohort design from a tertiary stroke center in Japan, and selection bias cannot be ruled out compared to nationwide studies covering a more diverse population. Second, the small sample size of subIS cases may lower the statistical power of our analysis. Third, we tried but could not include all possible prognostic variables of futile recanalization after EVT (e.g., collateral status, dementia, and proposed laboratory biomarkers) [6,27,28]. In addition, we did not consider stroke subtypes such as onset of stroke and TOAST classification because of the small number of patients (Table 1), which may also affect futile recanalization. Finally, the precise location of the subinsular cortex that may affect image analysis remains uncertain. The development of the image analysis tools that enable automated ROI segmentation and/or microstructural analysis would be expected. Despite these shortcomings, data from the current study can facilitate our understanding of post-EVT subIS and its outcome. It should be noted here that the involvement of subIS may increase the risk for futile recanalization, but this does not mean overall futility of EVT because there are no studies comparing the control group without a reperfusion attempt. Future randomized trials with a larger sample size including multiple prognostic variables are required to validate and expand these findings. 

## 5. Conclusions

Subinsular ischemic lesions contribute to poor functional independence in patients after the successful recanalization of left M1 occlusion. The attenuation of the ADC value, in addition to the physician’s visual assessment of DWI signals, in these territories on initial MRI could be a valuable predictor of therapeutic effectiveness. For future directions, the development of medical/endovascular strategies to restore ischemia extending to the subinsular cortex from the level of perforating arterial and/or collateral blood supply should be expected. Our findings may also highlight the importance of early and targeted rehabilitation therapy, particularly focusing on the neurological symptoms of subIS.

## Figures and Tables

**Table 1:** Clinical characteristics between patients with and without subIS.

Variables	Total (n = 35)	subIS (n = 11)	No-subIS (n = 24)	*p* Value
Age, median (IQR)	78 (72–86)	81 (78–89)	75 (72–83)	0.04 *
Female gender, n (%)	14 (40)	3 (28)	11 (46)	0.25
Hypertension, n (%)	22 (63)	7 (64)	15 (63)	0.62
Diabetes mellitus, n (%)	10 (29)	5 (45)	5 (21)	0.13
AF, n (%)	12 (34)	3 (27)	9 (38)	0.42
Premorbid mRS, median (IQR)	0 (0–0)	0 (0–0)	0 (0–0)	0.94
Baseline SBP, mean (SD)	161.0 (19.8)	162.4 (21.6)	160.3 (19.4)	0.65
Baseline NIHSS, median (IQR)	17 (12–23)	19 (18–24)	15 (12–22)	0.09
Unknown onset stroke, n (%)	10 (29)	3 (27)	7 (29)	0.62
Wake-up stroke, n (%)	3 (9)	2 (18)	3 (13)	
Daytime-unwitnessed stroke, n (%)	7 (20)	1 (9)	4 (16)	
ASPECTS, median (IQR)	9 (8–10)	9 (7–9)	9 (8–10)	0.32
ADC value, mean (SD)	0.59 (0.11)	0.52 (0.08)	0.62 (0.10)	0.006 *
Treatment with IV alteplase, n (%)	18 (51)	6 (55)	12 (50)	0.55
Time intervals, mean (SD) minutes				
OTD	261 (345)	246 (195)	268 (394)	0.21
OTR	363 (342)	295 (83)	388 (396)	0.52
PTR	48 (26)	43 (26)	51 (26)	0.18
First pass, n (%)	15 (43)	6 (55)	9 (38)	0.28
Stent retriever, n (%)	29 (85)	9 (82)	20 (83)	0.63
TOAST, n (%)				
Large artery atherosclerosis	2 (6)	0 (0)	2 (8)	
Cardioembolism	27 (77)	9 (82)	18 (75)	
Other or undetermined	6 (25)	2 (18)	4 (17)	
sICH, n (%)	2 (6)	1 (9)	1 (4)	0.54
ENI, n (%)	14 (40)	3 (27)	11 (46)	0.25
Three-month mRS, median (IQR)	2 (2–4)	4 (3–5)	2 (1–3)	<0.001 *
Futile recanalization (mRS ≥ 3), n (%)	17 (49)	9 (81)	8 (33)	0.01 *

Data are presented as mean (SD), median (IQR), or number (%). * *p* < 0.05, significant in univariate analysis. ADC, apparent diffusion coefficient; ASPECTS, Alberta Stroke Program Early CT score; AF, arterial fibrillation; ENI, early neurological improvement; IV, intravenous injection; NIHSS, National Institutes of Health Stroke Scale; mRS, modified Rankin Scale; OTD, onset-to-door; OTR, onset-to-recanalization; PTR, puncture-to-recanalization; SBP, systolic blood pressure; sICH, symptomatic intracerebral hemorrhage; subIS, subinsular stroke; TOAST, Trial of Org 10,172 in Acute Stroke Treatment.

**Table 2:** Multivariate binary logistic regression analysis of predictors for clinical outcomes.

Variables	aOR	95% CI	*p* Value
Age	0.99	0.91–1.08	0.85
NIHSS on admission	1.13	0.98–1.30	0.09
SubIS involvement	10.52	1.53–72.3	0.017 *

* *p* < 0.05, significant in multivariate analysis. CI, confidence interval; aOR, adjusted odds ratio by age; NIHSS, National Institutes of Health Stroke Scale; subIS, subinsular stroke.

## Data Availability

The datasets generated and analyzed during the present study are available from the corresponding author upon reasonable request. The data are not publicly available due to privacy and ethical restrictions.

## Supplementary Materials

The following supporting information can be downloaded at https://www.mdpi.com/article/10.3390/brainsci14090885/s1: Supplementary File S1 (Representative cases of subIS [restricted or with adjacent lesion] and non-subIS: Figures S1–S3), File S2 (Examples of ROI analyses: Figures S4–S6), and File S3 (Examples of functional MRI: Figure S7).
