# Supplementary material for: Involvement of Subinsular Territory Stroke as Predictor of Outcome after Successful Endovascular Recanalization of Left Middle Cerebral Artery Occlusion
Source: Brain Sci. 2024 Aug 30;14(9):885. doi: 10.3390/brainsci14090885 (PMC11430780; doi:10.3390/brainsci14090885)
Supplement: Supplementary file 1 [file brainsci-14-00885-s001.zip › Supplementary_Data_1_Fis_S1-S3.pdf]

Supplementary Data File 1

Case 1: subIS

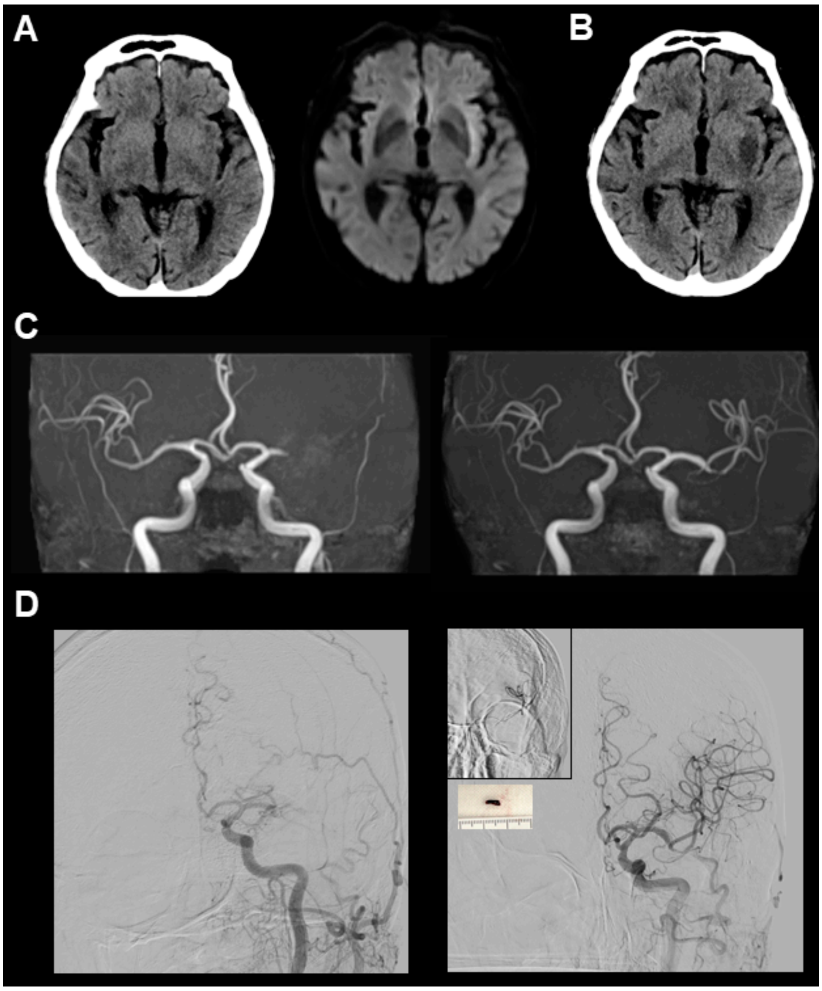

Figure S1. A representative case of subinsular stroke in an 81 year-old man admitted to our ER with right hemiparesis,d and total aphasia (NIHSS score of 17 and ASPECTS of 8). (**A**) CT scan (left) and diffusion-weighted MRI (right) on admission. (**B**) CT scan at one-week after EVT. Note that high signal intensities can be found on initial MRI along with the subcortical white matter restricted to the subinsular territory, suggestive of acute subIS, which was confirmed by low density area on the follow-up CT scan. (**C**) MR angiography and (**D**) DSA before (left) and after (right) EVT for left M1 occlusion. *Inset*, microcatheter contrast injection to confirm its position distal to the cot within a patent M2 branch and thrombus removed by EVT. A left M1 occlusion has been recanalized successfully on the first thrombectomy pass (mTICI 3). Although the patient's hemiparesis gradually improved up to assisted walking with a leg brace, transcortical aphasia and executive control deficits (e.g., swallowing and chewing) hindered his functional independence in the chronic stage. With a mRS score of 4, the patient was discharged to a nursing home 90 d after onset.

Case 2: subIS with adjacent ischemic lesion

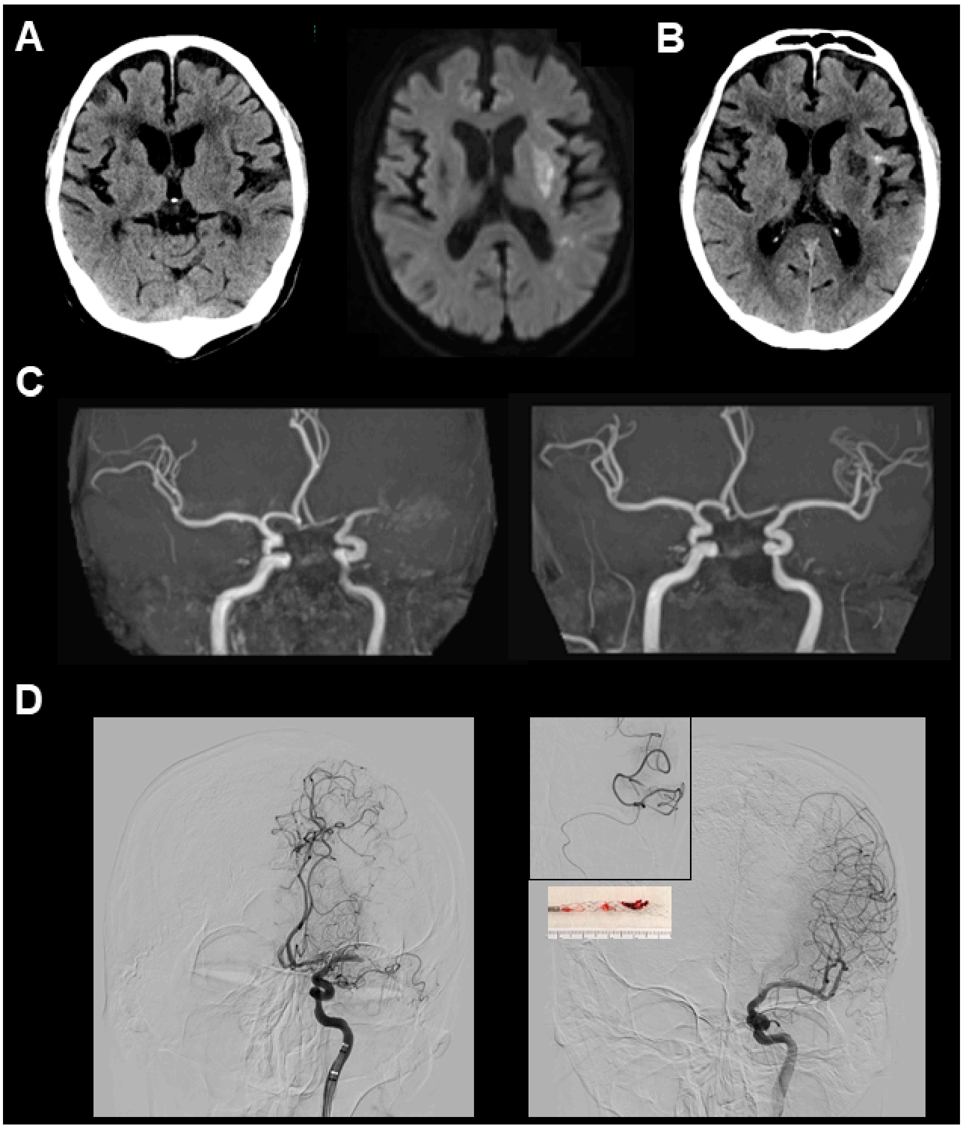

Figure S2. A representative case of subinsular stroke in a 70 year-old man admitted to our ER with right hemiparesis, right hemifacial palsy, and aphasia (NIHSS score of 15 and ASPECTS of 10). (**A**) Head CT (left) and brain diffusion-weighted MRI (right) on admission. (**B**) CT scan at one-week after EVT. High signal intensities along with the left subcortical white matter adjacent laterally to the putamen can be observed for initial MRI, suggestive of acute subIS, which was confirmed by low-density areas in subinsular cortex and putamen on the follow-up CT scan. (**C**) MR angiography and (**D**) DSA before (left) and after (right) EVT for left M1 occlusion. *Inset*, microcatheter contrast injection to confirm its position distal to the cot within a patent M2 branch and thrombus removed by a stent retriever. A left M1 occlusion has been recanalized successfully on the first thrombectomy pass (mTICI 3). The patient's walking ability and facial palsy gradually recovered. However, due to persisted motor aphasia and decreased executive function, the patient was discharged with a three-month mRS score of 3 in need of home healthcare services.

Case 3: No-subIS

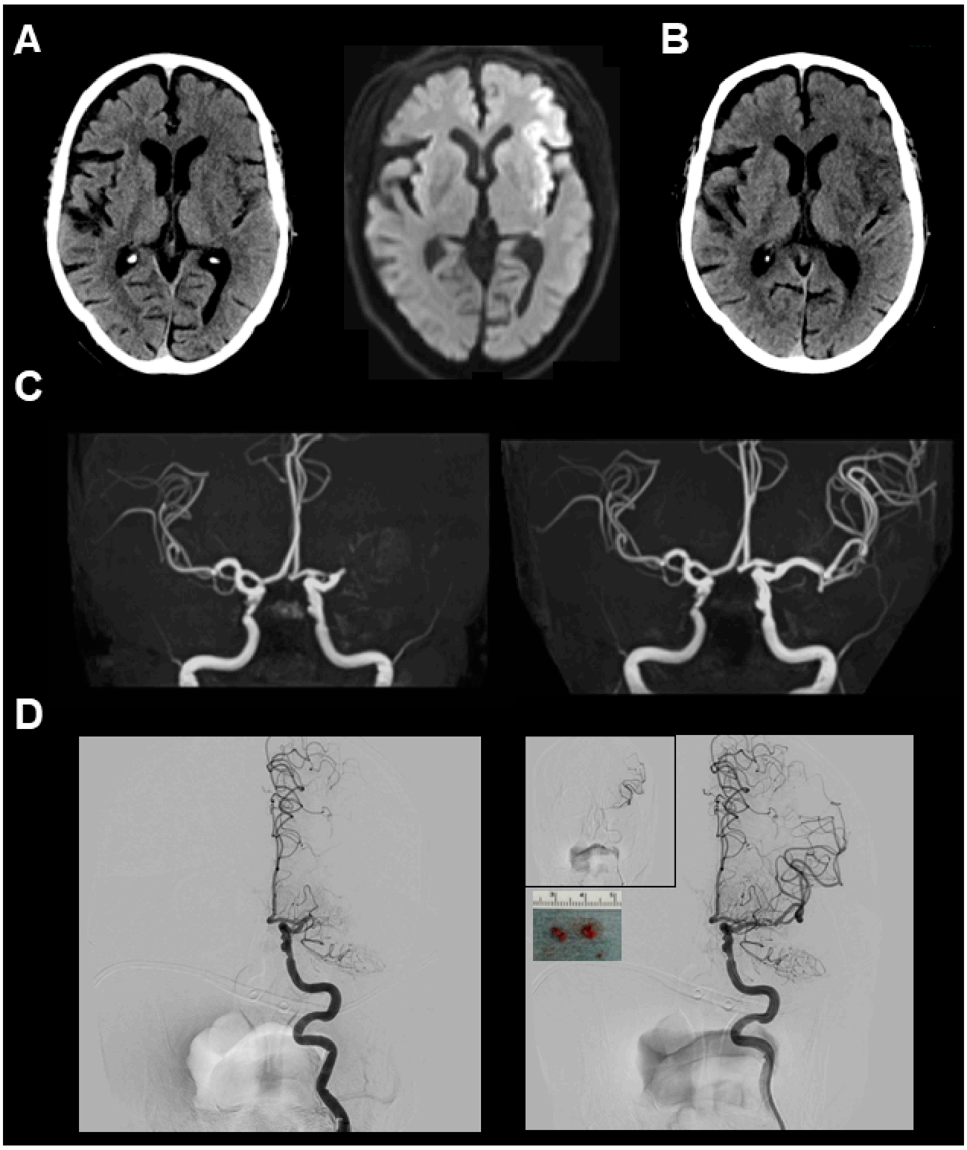

Figure S3. A representative case of no-subinsular stroke in an 88 year-old man admitted to our ER with right hemiparesis and total aphasia (NIHSS score of 18 and ASPECTS of 6). (**A**) CT scan (left) and diffusion-weighted MRI (right) on admission. (**B**) CT scan at one-week after EVT. High signal intensities can be seen on MRI along with the cortical white matter, suggestive of acute insular stroke, which was confirmed by peri-Sylvian low density area on the follow-up CT scan. (**C**) MR angiography and (**D**) DSA before (left) and after (right) EVT for left M1 occlusion. *Inset*, microcatheter contrast injection to confirm its position distal to the clot within a patent M2 branch and thrombus removed by EVT. A left M1 occlusion has been recanalized successfully on the first thrombectomy pass (mTICI 3). His hemiparesis improved early after EVT. The patient gradually recovered his speech ability with preserved cognitive functions, and was discharged to his home with mRS 2 at three months.
