# Supplementary material for: Involvement of Subinsular Territory Stroke as Predictor of Outcome after Successful Endovascular Recanalization of Left Middle Cerebral Artery Occlusion
Source: Brain Sci. 2024 Aug 30;14(9):885. doi: 10.3390/brainsci14090885 (PMC11430780; doi:10.3390/brainsci14090885)
Supplement: Supplementary file 1 [file brainsci-14-00885-s001.zip › Supplementary_Data_2_Figs_S4-S6.pdf]

Supplementary Data File 2

Examples of ROI analyses for cases 1–3 (*See* supplementary data file 1 for patient details)

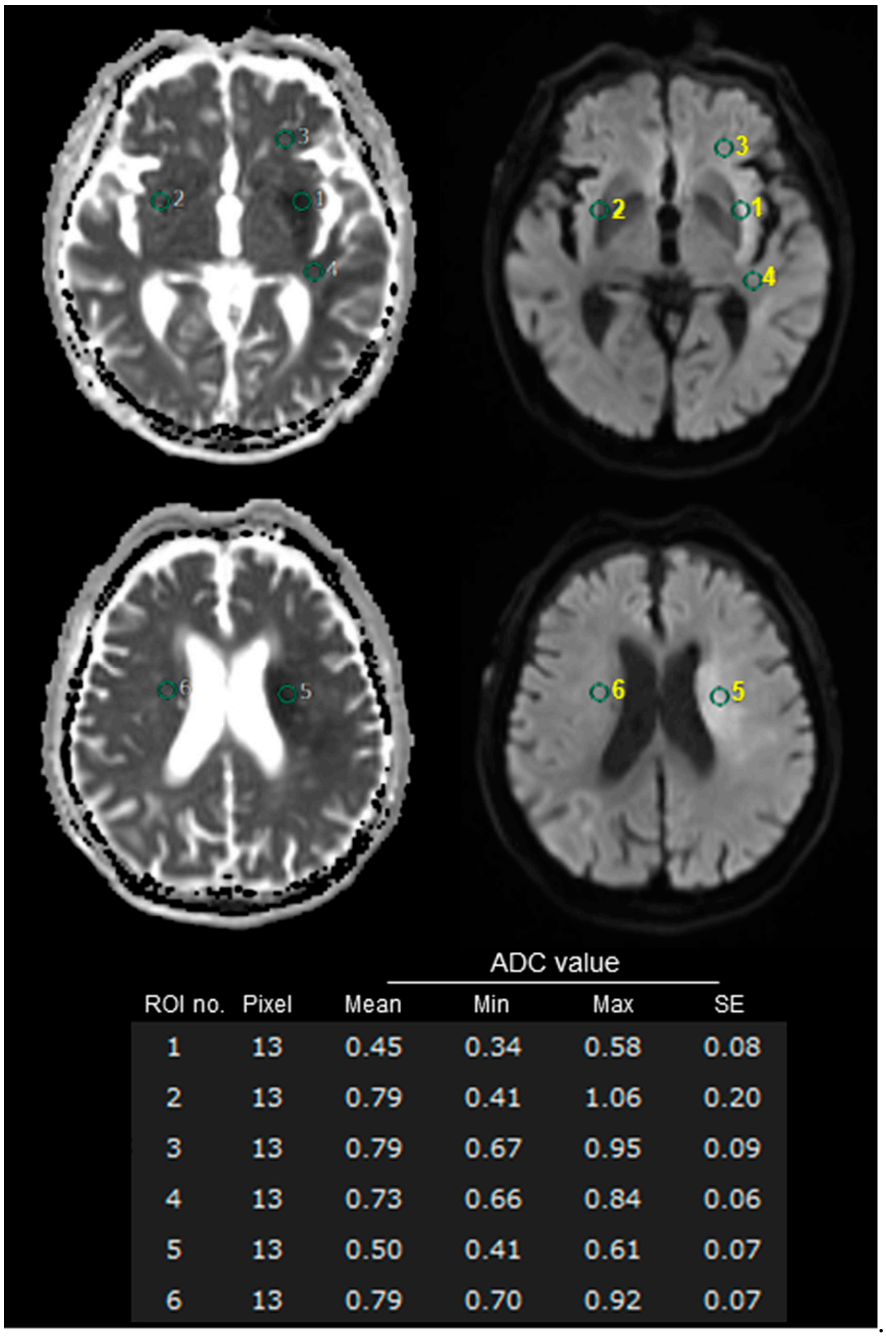

ROI no.	Pixel	ADC value Mean	Min	Max	SE
1	13	0.45	0.34	0.58	0.08
2	13	0.79	0.41	1.06	0.20
3	13	0.79	0.67	0.95	0.09
4	13	0.73	0.66	0.84	0.06
5	13	0.50	0.41	0.61	0.07
6	13	0.79	0.70	0.92	0.07

Figure S4. ROI analysis for Case 1: SubIS.

Six ROIs were selected from the center of the infarcted area (no.1), adjacent subcortical and deep white matter (nos.3, 4, and 5), and contralateral areas (nos.2 and 6) on the ADC figures with 13 pixels for each ROI to compute the average ADC value of the infarcted and reference regions. Note that the lower ADC values in the subinsular ischemic lesion (no.1) extended upward to the deep white matter around the slice level of the corona radiata (no.5). ADC, apparent diffusion coefficient; DWI, diffusion-weighted MR; ROI, region of interest.

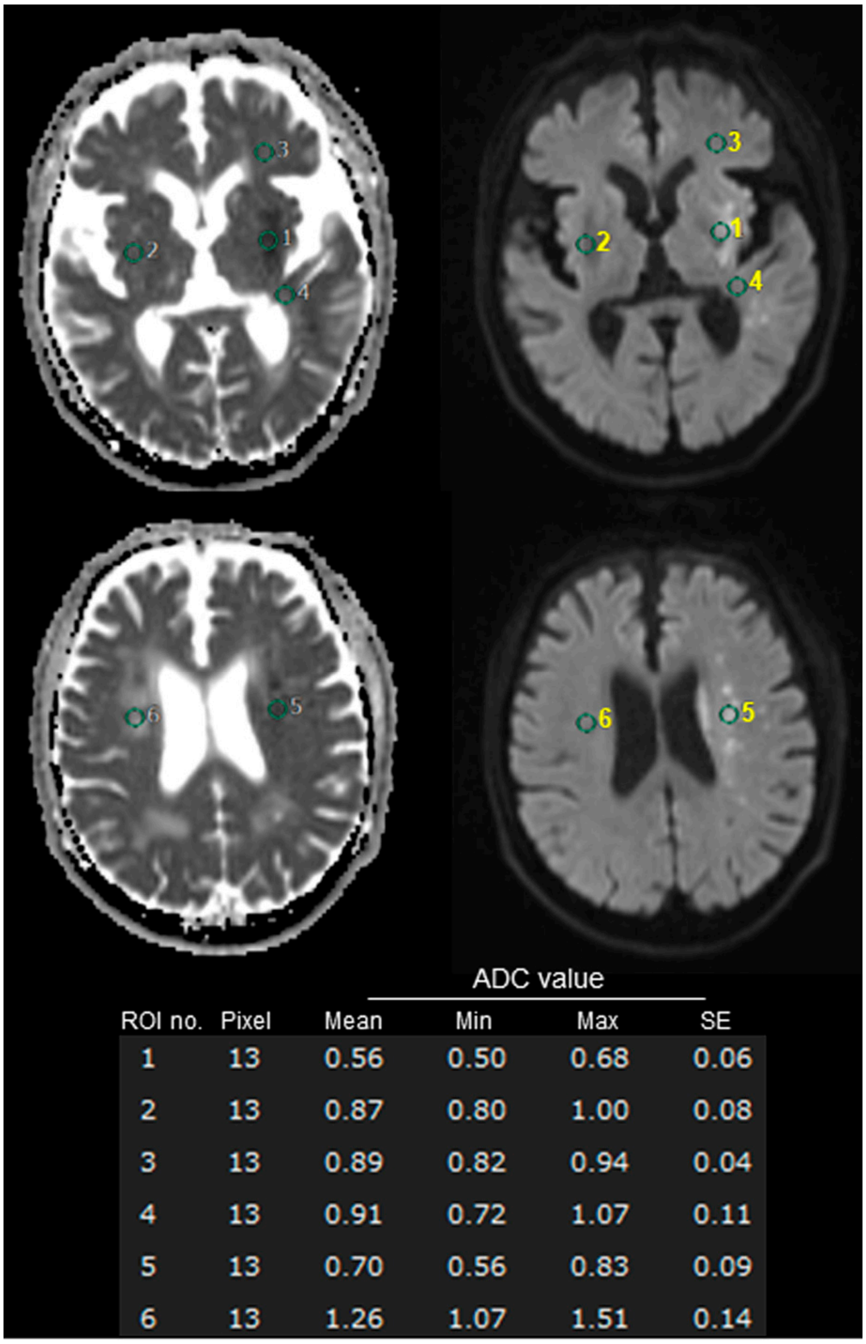

ROI no.	Pixel	ADC value Mean	Min	Max	SE
1	13	0.56	0.50	0.68	0.06
2	13	0.87	0.80	1.00	0.08
3	13	0.89	0.82	0.94	0.04
4	13	0.91	0.72	1.07	0.11
5	13	0.70	0.56	0.83	0.09
6	13	1.26	1.07	1.51	0.14

Figure S5. ROI analysis for Case 1: SubIS with adjacent cortical involvement.

Six ROIs were selected from the center of the infarcted area (no.1), adjacent subcortical and white matter (nos. 3, 4, and 5), and contralateral areas (nos. 2 and 6) on the ADC and DWI figures with 13 pixels for each ROI to compute the average ADC value of the infarcted and reference regions. The deep infarcts extending to the subinsular region (No.1) simultaneously showed sparse internal border zone infarcts at the slice level of the corona radiata (No.5). ADC, apparent diffusion coefficient; DWI, diffusion-weighted MR; ROI, region of interest.

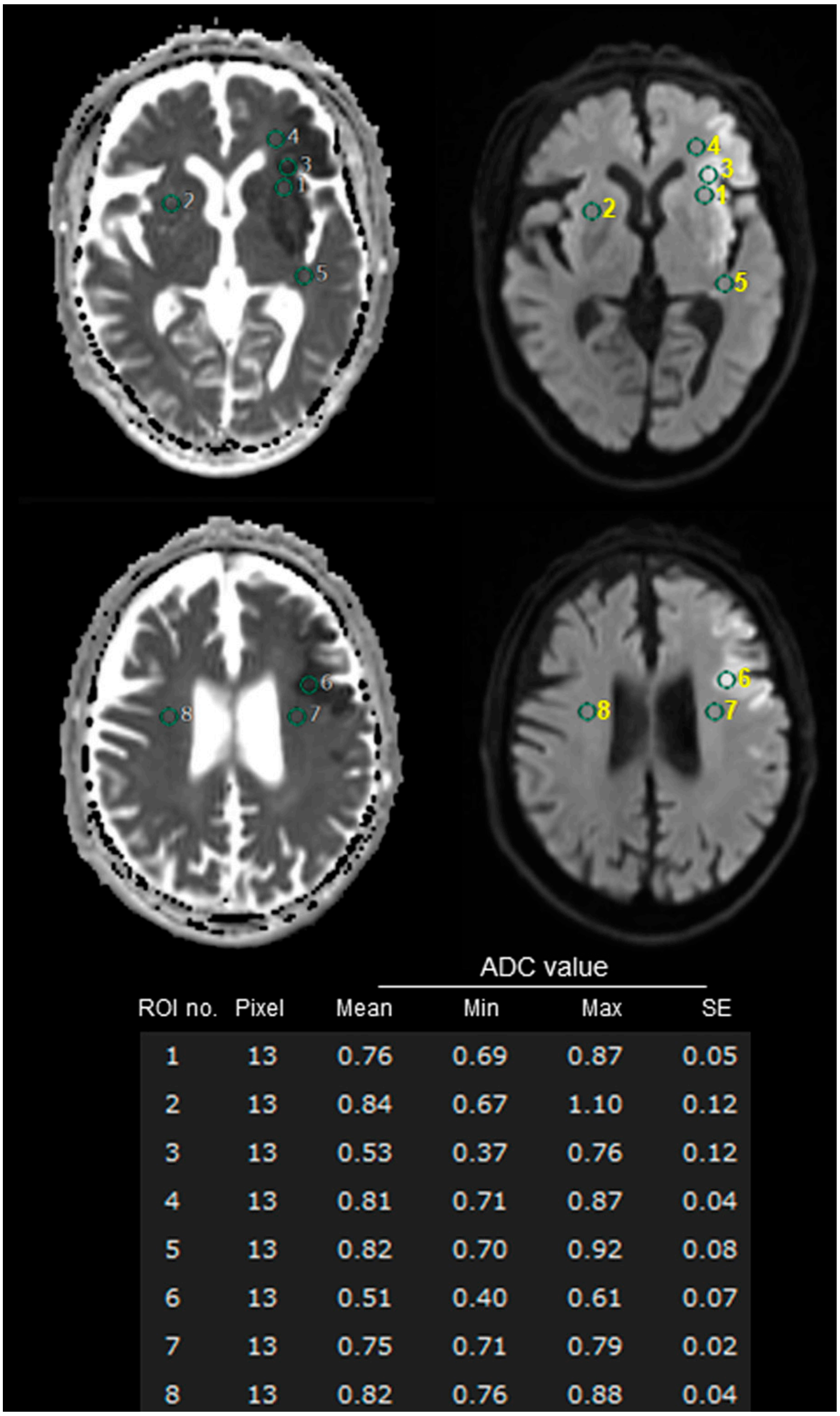

ROI no.	Pixel	ADC value Mean	Min	Max	SE
1	13	0.76	0.69	0.87	0.05
2	13	0.84	0.67	1.10	0.12
3	13	0.53	0.37	0.76	0.12
4	13	0.81	0.71	0.87	0.04
5	13	0.82	0.70	0.92	0.08
6	13	0.51	0.40	0.61	0.07
7	13	0.75	0.71	0.79	0.02
8	13	0.82	0.76	0.88	0.04

Figure S6. ROI analysis for case 3: No subIS.

Eight ROIs were selected from the center of the cortical infarcted area (nos. 2 and 6), adjacent subcortical and deep white matter (nos. 1, 4, 5, and 7), and contralateral areas (nos. 2 and 8) on the ADC and DWI figures with 13 pixels for each ROI to compute the average ADC value of the infarcted and reference regions. Note that the insular cortical ischemic lesion did not extend to the subcortical white matter (nos. 1 and 7). ADC, apparent diffusion coefficient; DWI, diffusion-weighted MR; ROI, region of interest.
