# Supplementary material for: Involvement of Subinsular Territory Stroke as Predictor of Outcome after Successful Endovascular Recanalization of Left Middle Cerebral Artery Occlusion
Source: Brain Sci. 2024 Aug 30;14(9):885. doi: 10.3390/brainsci14090885 (PMC11430780; doi:10.3390/brainsci14090885)
Supplement: Supplementary file 1 [file brainsci-14-00885-s001.zip › Supplementary_Data_3_Fig S7.pdf]

Supplementary Data File 3

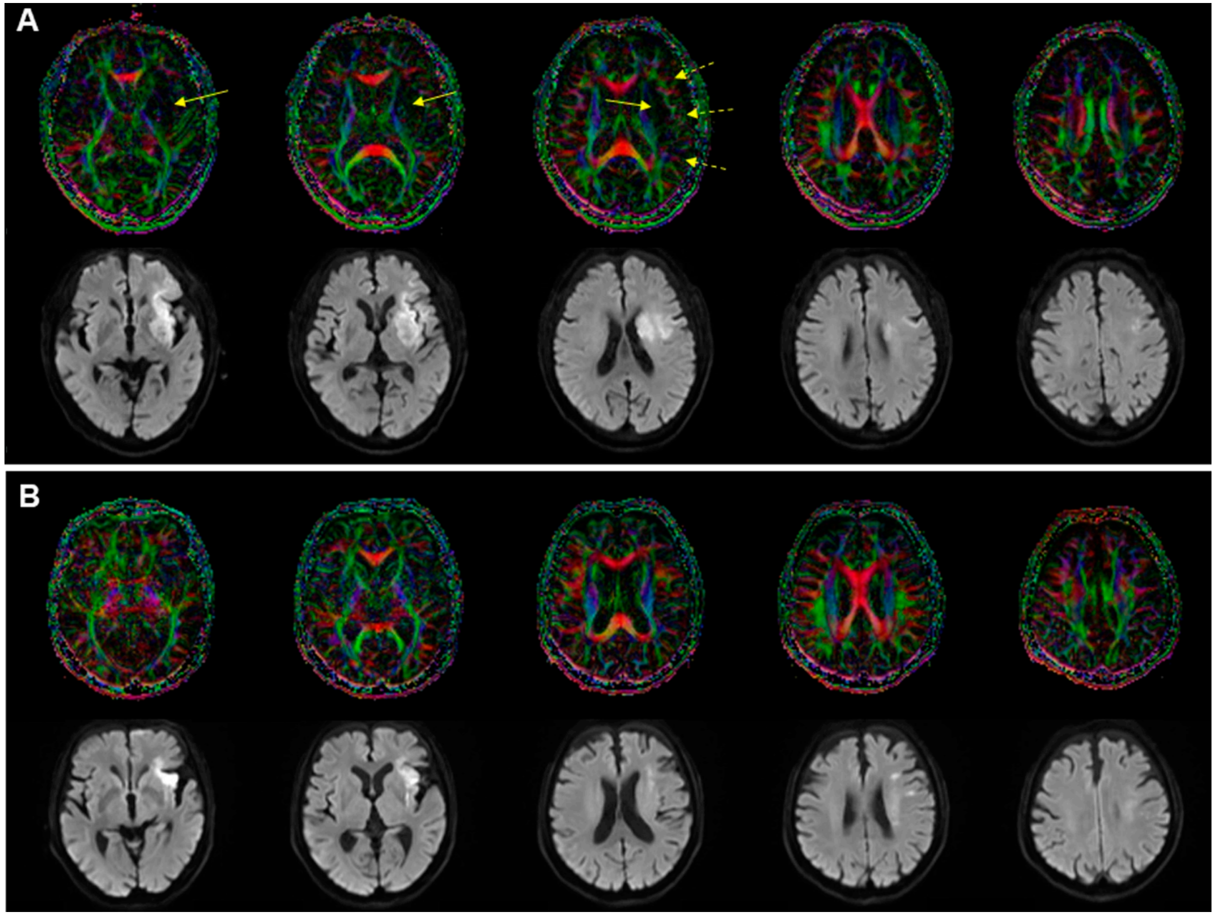

Figure S7. Examples of three-dimensional anisotropy contrast (3DAC, upper panels) and diffusion-weighted (DWI, lower panels) MRI in patients with (A) and without (B) subIS involvement after recanalization of the left M1 occlusion. Acute ischemic lesion can be visualized by high signals on DWI. 3DAC is an algorithm for the treatment of apparent diffusion tensor using the three primary colors (x, y, and z axis correspond to red, green, and blue color, respectively). Note that the patient with subIS showed distinct color fading in the subinsular territories (arrows) lateral to the pyramidal tract fibers (blue colors) with slight reduction of the association fibers in the corona radiata (dotted arrows). In no-subIS case, distinct color fading was observed around the insular cortex but no visible changes detected in deep cortical areas.
